# Quality of life is lower in food allergic adolescents compared to young children at a community educational symposium

**DOI:** 10.1186/s13223-023-00853-9

**Published:** 2023-11-27

**Authors:** Diem-Tran I. Nguyen, Kathleen Pitts, Kristen A. Staggers, Carla M. Davis

**Affiliations:** 1 Department of Pediatrics, Baylor College of Medicine, Houston, TX USA; 2 Division of Immunology, Allergy and Retrovirology, Baylor College of Medicine and Texas Children’s Hospital, Houston, TX USA; 3 Institute for Clinical and Translational Research, Baylor College of Medicine, Houston, TX USA

**Keywords:** Food allergy, Pediatrics, Quality of life

## Abstract

**Introduction:**

Food allergies (FA) can detrimentally impact physical, emotional, and psychological quality of life (QoL) among pediatric patients. Given the changes from childhood into adolescence, the impact of FA on QoL likely evolves with age. The purpose of this study was to determine whether QoL differed between adolescents and children with FA who participated in a Food Allergy Symposium (FAS).

**Methods:**

Patients with confirmed FA were recruited at an educational community symposium in September 2018 and September 2019. Patients and/or their parents were invited to complete the Food Allergy Quality of Life Questionnaires (FAQLQ). The Food Allergy Independent Measure (FAIM) reflects concerns about accidental food exposure and disease severity. Higher FAIM and FAQLQ scores reflect worse QoL. Summary scores were compared using the Wilcoxon rank sum test, Fisher’s exact test, or the Chi-square test.

**Results:**

Seventy-four surveys (82% children, 18% adolescents) were included. The FAQLQ total score was higher among adolescents than children (median 5.2 vs 4.2; p = 0.045), and the FAIM was lower in adolescents (median 2.2 vs 2.8; p = 0.037). More adolescents reported previous anaphylaxis than children (91.7% vs 51.8%; p = 0.011). The percentage reassured by having epinephrine was higher in adolescents (81.8% vs 45.8%; p = 0.046). No other QoL scores and survey responses were significantly different.

**Discussion:**

In this study, adolescents were more concerned about their disease and more reassured by epinephrine carriage than younger children, which may reflect increased autonomy and responsibility. Community events are an important way to assess QoL and provide FA-related education to pediatric patients.

## Background

Food allergies (FAs) have become increasingly common, affecting up to 4% of children and adolescents in the United States [1]. Studies have demonstrated that FA have a significant impact on physical, emotional, and psychological components of quality of life (QoL) [2, 3]. Strict avoidance of allergenic food is imperative, but can be difficult for pediatric patients and their parents. The fear of a grave reaction upon unintentional ingestion can heighten anxiety and lead to limitations in everyday activities such as attending school or going to a restaurant [2, 4].

Given the dramatic physical and emotional changes from childhood into adolescence, the impact of FA on QoL likely evolves with age. However, there is limited data on how QoL changes from 0 to 18 years of age. Prior studies surveying FA pediatric patients seen in AI clinic settings suggested that QoL was worse in teenagers (ages 13–17) compared to children (ages 0–12) [5, 6]. Furthermore, FA was shown to significantly affect family members, highlighting the need for education of patients and caregivers. Since participants at educational symposia might be more engaged in the management of their disease compared to a general AI clinic FA population, the purpose of this study was to determine whether QoL differed between FA adolescents and children who participated in an educational Food Allergy Symposium from 2018 to 2019.

## Methods

The Food Allergy Symposium (FAS) is an educational conference hosted by the Baylor College of Medicine (BCM) for FA children and their family members. Attendees were invited to complete the Food Allergy Quality of Life Questionnaire (FAQLQ), a validated instrument that assesses QoL in FA families [7, 8].

Children and adolescents ages 0–17 years old with a diagnosis of FA were recruited at the FAS hosted by the BCM in September 2018 and 2019. FA adolescents, children, and their parents were invited to complete the FAQLQ. Parents completed the FAQLQ-Parent Form (FAQLQ-PF) for children 0–12 years of age, while adolescents 13–17 years of age completed the FAQLQ-Teenager Form (FAQLQ-TF) independently. Participants provided informed consent and data was collected anonymously. This study was approved by the BCM institutional review board.

The FAQLQ consists of multiple subscales for various domains of FA such as social and dietary limitations (SDL), emotional impact (EI), and food anxiety. These subscale scores can be combined to obtain an overall mean FAQLQ score. In addition, the Food Allergy Independent Measure (FAIM) score, which reflects concerns about accidental food exposure and disease severity, can also be obtained from the FAQLQ surveys [7, 8]. Higher FAIM and FAQLQ scores reflect worse QoL.

Patient characteristics and survey scores were summarized by median with minimum and maximum value, or frequency with percentage. Comparisons between children and adolescents were made using the Wilcoxon rank sum, Fisher's exact or Chi-square test.

## Results

A total of 74 patients participated in the study, with over 80% (n = 61) ages 0–12 years and the remainder adolescents. In summary, the median age of participants was 9 years old, and median number of food allergies was 2.5. (Table 1) The most common FA was peanut (81.4%, n = 57/70). Fifty-nine percent (n = 40/68) of participants reported previous anaphylaxis and 94.3% (n = 66/70) of participants had an epinephrine auto-injector.

**Table 1:** Summary Statistics

	N total	N (%)		
Year				
2018	74	51 (68.9)		
2019	74	23 (31.1)		
Child sex				
F	70	34 (48.6)		
M	70	36 (51.4)		
			Median	(Min, Max)
Child age (years)	74	70	9 [0.5,17.0]	[0.5,17.0]
Number of symptoms	74	71	7 [0.0,27.0]	[0.0,27.0]
FAQLQ: Emotional impact score	74	67	4 [1.0,6.8]	[1.0,6.8]
FAQLQ: Food anxiety score (child only)	74		4.3	[1.0,7.0]
FAQLQ: Social and dietary limitations score	74		4.7	[1.1,7.0]
FAQLQ: Food allergy health score (teen only)	74		5.3	[3.3,7.0]
FAQLQ: Risk score (teen only)	74		5	[2.8,7.0]
	74		4.3	[1.0,6.9]
Food allergy independent measure (FAIM)	74	70	2.8	[0.6,5.4]

### Comparison between children and adolescents

While the median number of FAs was not statistically different between children and adolescents (3 vs 2, p = 0.084), children were more likely to have more than 6 FAs compared to adolescents (31.5% vs 0%, p = 0.027) (Table 2). The FAQLQ total score was significantly higher among adolescents than children (median 5.2 vs 4.2; p = 0.045), reflecting a lower QOL. The FAIM was lower in adolescents than children (median 2.2 vs 2.8; p = 0.037). More adolescents reported previous anaphylaxis (91.7% vs 51.8%; p = 0.011) and feeling reassured by having epinephrine (81.8% vs 45.8%; p = 0.046). Adolescents also had a higher percentage of dysphagia (58.3% vs 22%; p = 0.029).

**Table 2:** Survey comparisons between children and adolescents

	Children (N = 61)		Adolescents (N = 13)		p-value^1^
	N	N (%)	N	N (%)	
Parent sex (female)	58	50 (86.2)	12	11 (91.7)	> 0.99
Child sex (female)	58	26 (44.8)	12	8 (66.7)	0.213
Greater than 6 FAs	54	17 (31.5)	12	0 (0)	0.027
	N	Median (Min, Max)	N	Median (Min, Max)	
FAQLQ total score	60	4.2 (1.0, 6.8)	11	5.2 (3.1, 6.9)	0.045
FAQLQ food anxiety score (child only)	59	4.3 (1.0, 7.0)	N/A	N/A	N/A

^1^Comparisons between categorical variables used Fisher’s exact or Chi-square test. Comparisons between continuous variables used Wilcoxon rank-sum test

## Discussion

In our study, in an engaged patient population at a food allergy educational symposium, adolescents had significantly lower QoL than children, reflected by higher FAQLQ scores despite having fewer FA. Teens generally reported more concern about their disease and more reassurance by epinephrine carriage than children, which may be due to their increased autonomy regarding their health. These findings underscore that engagement in food allergy symposia does not dilute the effect shown in prior studies demonstrating that older children, ages 6–12 years, have worse QoL compared to children 0–5 years [6]. Miller et al*.* also found that increased age was associated with worse SDLs and EI [5]. As children mature and have less parental supervision, they have greater personal responsibility for their health which may lead to poorer QoL [5, 6].

FA teenagers have reported struggling in school, depression, and social isolation as a result of their disease [4, 7]. As teenagers mature, they have greater appreciation of the reality of having a lifelong disease [3, 4]. In addition, the need to create and maintain a FA free environment can make teens feel singled out [7]. For example, up to 24% of FA teenagers and adults report being bullied for their FA at some point during their life, including intentional contamination of their lunches with their allergenic food [3, 4]. Having a chronic condition that requires constant vigilance during a time of intense physical and emotional growth likely worsens QoL, which may explain the difference seen in our study between children and teenagers.

Despite lower QoL scores, in our study, adolescents had lower FAIM scores reflecting less anxiety about accidental food exposure. This may be due to more engagement in educational opportunities, increased experience at avoiding allergenic foods, or differences in parental and adolescent perceptions of risk of unintentional allergen ingestion. Furthermore, adolescents in this study had less FA than children, decreasing the burden of needing to avoid multiple foods. Given the influence of FA on patient and family QoL, education on FA may decrease anxiety. Group FA education and training has been shown to improve parental QoL [2]. Providing families with tools to manage FA can foster coping mechanisms.

The higher incidence of dysphagia in teenagers may indicate eosinophilic esophagitis (EoE) as an under-recognized cause of dysphagia among FA youth. This may have contributed to a worse QoL. Dysphagia is a common manifestation of EoE in older children. EoE is a FA-associated chronic disease with inflammatory responses to food antigen, and > 90% of EoE patients will respond to an elimination diet that avoids identifiable trigger foods. Symptoms of the disease often recur with re-introduction of the identified trigger foods. The incidence of EoE in FA pediatric patients has been reported to be higher than in the general population with estimates of around 100-fold higher than the non-FA population [9]

Our study has several limitations. First, our participants were recruited at an educational symposium, which may select for patients who are more concerned about their FA. Second, there were a small number of adolescent patients. Also, there may be differences between adolescents and children due to parental reports (proxy report) for young children.

## Conclusion

FA have a significant impact on patients and their families. This assessment of QoL and FA symptoms at a community educational event reveals a strong impact of FA in adolescent populations compared to children. This difference may be due to increased autonomy and better understanding of their illness. Empowering patients and their families with skills needed to manage their FA is associated with decreased anxiety. Community educational symposiums can serve as avenues to disseminate coping skills to patients and their families. Further investigation is warranted to better understand how to best address factors that contribute to worsening QoL.

## Data Availability

The data generated and analyzed during this study are not publicly available due to institutional policies but are available from the corresponding author on reasonable request and with permission from Baylor College of Medicine.

## References

[1] Gupta RS, Warren CM, Smith BM (2019). The public health impact of parent-reported childhood food allergies in the United States. Pediatrics.

[2] Walkner M, Warren C, Gupta RS (2015). Quality of life in food allergy patients and their families. Pediatr Clin North Am.

[3] Feng C, Kim JH (2019). Beyond avoidance: the psychosocial impact of food allergies. Clin Rev Allergy Immunol.

[4] Cummings AJ, Knibb RC, King RM, Lucas JS (2010). The psychosocial impact of food allergy and food hypersensitivity in children, adolescents and their families: a review. Allergy.

[5] Miller J, Blackman AC, Wang HT, Anvari S, Joseph M, Davis CM, Staggers KA, Anagnostou A (2020). Quality of life in food allergic children: results from 174 quality-of-life patient questionnaires. Ann Allergy Asthma Immunol.

[6] Thörnqvist V, Middelveld R, Wai HM, Ballardini N, Nilsson E, Strömquist J, Ahlstedt S, Nilsson LJ, Protudjer JLP (2019). Health-related quality of life worsens by school age amongst children with food allergy. Clin Transl Allergy.

[7] Kachru R (2020). Psychosocial issues and quality of life associated with food allergy. J Food Allergy.

[8] de Blok BMJF, Flokstra-de Blok BM, Dubois AEJ (2012). Quality of life measures for food allergy. Clin Exp Allergy.

[9] Wilson JM, Li RC, McGowan EC (2020). The role of food allergy in eosinophilic esophagitis. J Asthma Allergy.

